# Nocturnal heart rate variability parameters as potential fibromyalgia biomarker: correlation with symptoms severity

**DOI:** 10.1186/ar3513

**Published:** 2011-11-16

**Authors:** Claudia Lerma, Aline Martinez, Natllely Ruiz, Angelica Vargas, Oscar Infante, Manuel Martinez-Lavin

**Affiliations:** 1 Electromechanical Instrumentation Department, National Institute of Cardiology Ignacio Chavez, Mexico. Juan Badiano 1, 14080. Mexico City, Mexico; 2 Rheumatology Department, National Institute of Cardiology Ignacio Chavez, Mexico. Juan Badiano 1, 14080. Mexico City, Mexico

## Abstract

**Introduction:**

At present, there is neither a laboratory test nor an imaging technique able to differentiate people with fibromyalgia (FM) from healthy controls. This lack of an objective biomarker has hampered FM recognition and research. Heart rate variability (HRV) analyses provide a quantitative marker of autonomic nervous system activity. Nighttime is a stable period in which most people are resting. Sleep is modulated by autonomic activity. Sleeping problems are prominent in FM. The objectives of this study are: 1) to explore different nocturnal HRV parameters as potential FM biomarkers and 2) to seek correlation between such HRV parameters and diverse FM symptoms.

**Methods:**

We studied 22 women suffering from FM and 22 age-matched controls. All participants filled out several questionnaires related to FM symptoms. All participants used a Holter monitor over 24 hours while undertaking their routine activities during the day and while sleeping at their homes at night. Time-domain HRV parameters analyzed from 0000 to 0600 hours included, among others: mean normal-normal interbeat intervals (mean NN), standard deviation of the NN intervals (SDNN), and standard deviation of the successive NN differences (SDSD).

**Results:**

Nocturnal SDNN of less than 114 ms had the greatest predictive value to set apart patients from controls with an odds ratio of 13.6 (95% confidence interval: 3.9 to 47.8). In patients, decreased nighttime HRV markers indicative of sympathetic predominance had significant correlations with several FM symptoms: SDSD was associated with pain intensity (r = - 0.65, *P *= 0.001). SDNN correlated with constipation (r = - 0.53, *P *= 0.001), and mean NN with depression (r = - 0.53, *P *= 0.001). Controls displayed an opposite behavior. For them, increased nighttime SDNN correlated with Fibromyalgia Impact Questionnaire scores (r = 0.69, *P *= 0.001) and with other FM symptoms.

**Conclusions:**

Nocturnal HRV indices indicative of sympathetic predominance are significantly different in FM women when compared to healthy individuals. In FM patients, these HRV parameters correlated with several symptoms including pain severity. Opposite associations were seen in controls. FM may not be just one end of a continuous spectrum of common symptoms. Nocturnal HRV analyses are potential FM biomarkers.

## Introduction

Fibromyalgia is a common controversial illness with prevalence in the general population ranging from 3 to 5%. The overwhelming majority of affected individuals are women. Patients who suffer from FM often have multiple complaints related to pain, sleep, fatigue, anxiety, and depression [1]. Some investigators conceive FM as just one end of a continuous spectrum of 'normal' symptoms [2]. Thus the label 'fibromyalgianess' has been proposed to describe this condition [3]. At present, there is neither a laboratory test nor an imaging technique able to set apart people who suffer from FM from healthy controls. This lack of objective marker has hampered FM recognition and research. A biomarker is defined as a characteristic that can be objectively measured and evaluated as an indication of normal or pathogenic processes or pharmacological responses to a therapeutic intervention [4,5].

A consistent line of research has shown that patients who suffer from FM have signs of autonomic dysfunction, specifically signs of relentless sympathetic activity accompanied by sympathetic hypo-reactivity to stress [6]. It has been proposed that such autonomic dysfunction is the cause of the multiplicity of FM symptoms and that FM is a sympathetically-maintained neuropathic pain syndrome [6]. Most studies looking for autonomic performance in FM have used heart rate variability (HRV) analysis as a probing instrument. Almost all of these HRV studies were done during daytime. Due to the rapid advances of computer-based science, HRV analysis is becoming a useful non-invasive clinical tool to study autonomic nervous system performance.

HRV analysis is based on the well-known fact that the heart rate is not fixed, but varies from beat to beat constantly. The antagonistic effects of the sympathetic and parasympathetic branches of the autonomic nervous system on the sinus node harmonize the periodic components of this constant variability. Heart rate variability can be studied in the time domain, where the basic units are milliseconds. In the context of sinus rhythm, more beat-to-beat variability of the heart rate signifies more parasympathetic impulses on the sinus node. In resting conditions, less overall HRV reflects sympathetic predominance on the sinus node. Sympathetic predominance means either higher sympathetic activity or decreased parasympathetic activity, or both [7]. Healthy young individuals have ample HRV, whereas different diseases as well as aging decrease HRV. After myocardial infarction, diminished HRV is a strong predictor of sudden death [8].

Preliminary genetic studies support FM dysautonomic nature. Gene variations of two key sympathetic elements have been described in this illness. FM patients have polymorphisms associated with a defective catechol-O-methyl- transferase (COMT) enzyme [9,10]. COMT is involved in the inactivation of catecholamines. FM patients also have gene variations associated with dysfunctional adrenergic receptors. These receptors modulate pain perception and orthostatic balance [11]. We are not aware of epigenetic studies in FM.

Patients with FM frequently have sleeping problems including increased awakenings and reduced slow wave sleep. EEG evidence of disturbed sleep was the first objective alteration found in FM [12]. The autonomic nervous system has a direct effect on sleep architecture [6]. Nighttime appears to be an ideal period to study autonomic performance through HRV analysis. During this period most people are in a stable basal supine position. They are asleep or trying to do so.

The present study had two objectives: 1) to explore different nocturnal HRV parameters as potential FM biomarkers and 2) to seek correlations between such HRV parameters and different FM symptoms, reported in validated questionnaires related to pain, autonomic dysfunction, fatigue, sleep, anxiety and depression.

## Materials and methods

### Participants

All participants were women. Eligibility criteria for patients were the following: 1) to have FM according to the 1990 American College of Rheumatology guidelines [13]; 2) to be free of any medication that could affect sleep and/or autonomic performance including tranquilizers or antidepressants; 3) to be 18 to 50 years old; 4) to be in the fertile period of their lives with active menstrual cycles, but not to be in their menstrual period the day of the study; 5) to have no comorbid conditions; and 6) to freely agree to participate in the study. Patients were sourced from different rheumatology private practices in Mexico City.

Eligibility criteria for controls were the following: 1) to consider themselves healthy and to have 5 or fewer FM tender points; and 2) Not to be in their menstrual period the day of the study. For each patient, a control of similar age (± 2 years) was recruited. Controls were medical or paramedical personnel. A rheumatologist examined all prospective participants to ascertain the diagnosis of FM or the healthy status of controls. This clinical assessment was considered the reference standard for the potential biomarker.

All participants signed a written consent form. The study was approved by the Research and Bioethics Committee of the National Institute of Cardiology of Mexico on August 7, 2009 (Reference number: PT-28).

### Setting and data collection

The day of the study, questionnaires and other data were prospectively collected at the Department of Electromechanical Instrumentation of the National Institute of Cardiology in Mexico City. Then, participants were connected to a portable Holter recorder. Twenty- four-hour Holter recordings were done while participants followed their routine activities during the day and while sleeping at their homes at night. Studies were done during working days (from Monday to Thursday),

### Test methods

#### Symptoms evaluation

All participants filled out validated Spanish versions of six questionnaires related to FM symptoms, autonomic dysfunction, sleep quality, anxiety, depression, fatigue, and general wellbeing. These six questionnaires were: Fibromyalgia Impact Questionnaire (FIQ) [14], Medical Outcome Sleep Scale (MOS) [15], Composite Autonomic Symptoms and Signs (COMPASS) [16], Hospital Anxiety and Depression Scale (HADS) [17], Multidimensional Assessment of Fatigue Scale (MAF) [18], and Health Survey Short Form-36 (SF-36) [19]. FIQ is an instrument designed to estimate the overall impact of fibromyalgia over many dimensions (for example, function, pain level, fatigue, sleep disturbance, and psychological distress). It is scored from 0 to 100 with the latter number being the worst case. The average score for patients seen in tertiary care settings is about 50. MOS measures six dimensions of sleep; initiation, maintenance, quantity, adequacy, somnolence, and respiratory impairment. Indexes are scored on a 0-100 possible range. Healthy people have MOS average count in the region of 29. COMPASS explores symptoms related to nine different autonomic function domains: orthostatic, secretomotor, male sexual dysfunction (not used in this study), urinary, gastrointestinal, pupillomotor, vasomotor, syncope, and sleep function. COMPASS also contains an understatement index and a psychosomatic index. The worst possible overall count for women is 170. Healthy individuals have mean COMPASS scores in the order of 10. HADS has 14 intermingled anxiety and depression items that score from 0 to 21. A HADS count of 11 or higher suggests the presence of a mood disorder. MAF is a 16 item scale that measures fatigue according to four dimensions: degree and severity, distress that it causes, timing of fatigue, and its impact on various activities of daily living. A 'normal' MAF score is about 17, the worst possible count is 50. SF-36 is a multi-purpose, short-form health survey with 36 questions. It yields an 8-scale profile of functional health and well-being indexes as well as psychometrically-based physical and mental health items. In contrast to all previous questionnaires, an SF-36 higher count indicates a better quality of life. The best possible tally is 100. A normal SF-36 mental component count is in the region of 50. The same normal score is also true for SF-36 physical components.

A Holter monitor (model DMS-307, DMS Inc.), was used for electrocardiogram recording. Beat identification and classification were performed by the automated computer program designed for these Holter recorders (Premier 11, Meigaoyi Inc.). Time intervals from ectopic beats were detected and substituted by interpolated values of normal RR intervals using an adaptive filtering method (available on: http://tocsy.agnld.uni-potsdam.de/) [20]. Beat-to-beat time series (HRV signal) were analyzed by a computer program produced and validated in our technological development laboratory [21]. Readings were done by an HRV analysis expert who was blinded to the participant clinical status. The following time-domain HRV parameters were analyzed: mean NN interval (mean NN), standard deviation of the NN intervals (SDNN), mean standard deviation of the average NN intervals calculated over 5 minutes (SDANN), standard deviation of the successive NN differences (SDSD), the square root of the mean of the sum of successive NN differences (RMSSD), and percentage of adjacent pairs of RR intervals that differed by more than 50 ms from each other (pNN50). SDNN and SDANN are indicative of overall HRV. SDSD, RMSSD and pNN50 evaluate beat-to-beat fluctuations. Although all the indexes are influenced by both sympathetic and parasympathetic activity, those extracted from beat-to-beat variability are considered good estimators of parasympathetic modulation of heart rate [7,22].

All HRV parameters were defined according to international standards of measurements [8]). Calculations were done for the entire day of the study and a sub-analysis was focused on sleeping hours (from 0000 hours to 0600 hours).

#### Sample size calculation

Calculation of sample size (N) was based on a previous study from our institution [23]. In that study, the difference in mean SDANN between FM patients and controls was 26 ms. The FM group's SDANN standard deviation was 37 ms. The control group's SDANN standard deviation was 31 ms. Considering an alpha error = 0.05 (Z_α _= 1.96), and beta error = 0.2 (Z_β _= 0.842), with a statistical power of 80% for bilateral test, then N was calculated as 22 subjects per group.

#### Statistical analysis

**Figure 1:**
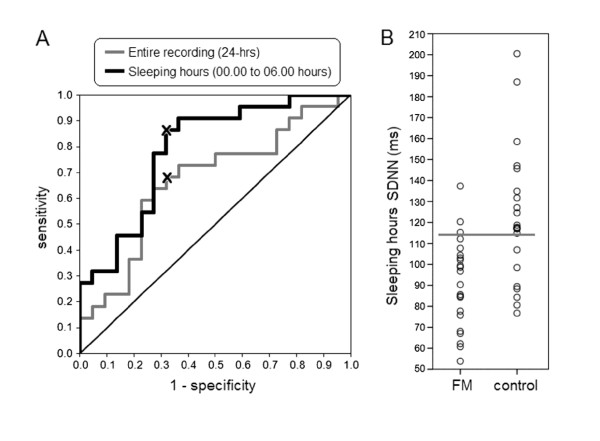
**Receiver-operator characteristics (ROC) curves (A) and scatter gram (B) of the nocturnal heart rate variability parameter SDNN, evaluated from Holter recording of 22 female patients with fibromyalgia and 22 matched healthy controls**. The best cut-off point (indicated by symbol 'x' in Figure 1-A was determined according to the shortest orthogonal distance from each point to the optimum value (0,1). The line in figure 1-B indicates the cut-off point (114 milliseconds (ms). SDNN, standard deviation of the NN intervals.

Clinical data are expressed as mean ± standard deviation. Normal distribution was confirmed by the Kolmorogv-Smirnov test. Differences between groups were analyzed by *t *test or Mann-Whitney U test according to distribution type. The best cut-off point for each parameter was determined from receiver-operator characteristic (ROC) curves, according to the shortest orthogonal distance of each curve point to the optimal point. An example is shown in Figure 1. Sensitivity, specificity, positive predictive value, negative predictive value and odds ratio were estimated for each parameter. Pearson's or Spearman's methods were used to search for correlations between HRV parameters and the severity of fibromyalgia symptoms. Statistical analysis was performed with version 16.0 of the SPSS computer program (StatSoft, Inc).

## Results

Patients and controls had similar demographic characteristics. In contrast, sharp differences in questionnaire's responses were recorded (Table 1).

**Table 1:** Demographic features and symptom scores in fibromyalgia patients and controls.

Variable	Fibromyalgia (n = 22)	Control (n = 22)	*P *value
Age (years)	32.4 ± 7.9	30.4 ± 7.4	0.406
Body mass index (kg/m2)	24.6 ± 4.4	25.0 ± 3.2	0.719
Disease duration (years)	6.8 ± 6.1	N/A	N/A
Total FIQ score	64.8 ± 14.1	12.0 ± 10.7	< 0.0001
VAS disability	6.5 ± 2.3	0.5 ± 1.1	< 0.0001
VAS pain	7.2 ± 2.2	0.7 ± 1.5	< 0.0001
VAS fatigue	8.3 ± 1.4	1.8 ± 2.2	< 0.0001
VAS sleep	7.3 ± 3.1	2.1 ± 2.4	< 0.0001
VAS morning stiffness	7.2 ± 2.5	0.7 ± 1.4	< 0.0001
VAS anxiety	7.3 ± 2.4	2.1 ± 2.3	< 0.0001
VAS depression	6.3 ± 3.1	1.8 ± 2.5	< 0.0001
Total COMPASS score	53.6 ± 18.2	13.5 ± 10.6	< 0.0001
Orthostatic intolerance	20.4 ± 9.6	5.2 ± 5.8	< 0.0001
Vasomotor domain	3.3 ± 2.9	0.4 ± 1.1	< 0.0001
Secretomotor	4.7 ± 3.6	1.0 ± 1.5	< 0.0001
Gastroparesis	2.9 ± 1.5	0.5 ± 0.7	< 0.0001
Diarrhea	7.4 ± 5.4	2.5 ± 3.1	0.001
Constipation	4.4 ± 2.4	1.1 ± 1.2	< 0.0001
Bladder	4.0 ± 4.7	1.0 ± 1.6	0.010
Pupillomotor	2.8 ± 1.2	0.7 ± 1.1	< 0.0001
Sleep	2.8 ± 1.7	0.5 ± 0.8	< 0.0001
Syncope	0.5 ± 1.4	0.1 ± 0.8	0.305
Understatement index	0.8 ± 1.3	3.0 ± 2.9	0.003
Psychosomatic index	1.2 ± 1.5	0.2 ± 0.6	0.004
MAF	40.3 ± 5.5	15.6 ± 11.3	< 0.0001
MOS	60.5 ± 11.2	19.2 ± 10.4	< 0.0001
SF-36 mental component	38.9 ± 5.3	42.2 ± 5.2	0.043
SF-36 physical component	53.0 ± 7.6	63.0 ± 83	< 0.0001
HADS depression	7.3 ± 3.0	3.9 ± 4.4	0.005
HADS anxiety	12.3 ± 4.0	5.5 ± 2.9	< 0.0001

COMPASS, composite autonomic symptom scale; FIQ, fibromyalgia impact questionnaire; HADS, hospital anxiety and depression scale; MAF, multidimensional assessment of fatigue; MOS, medical outcome sleep scale; SF-36, short form 36 items health survey; VAS, visual analogue scale. N/A = not applicable.

HRV results evaluated from the entire recording, including those obtained during sleeping hours, are shown in Table 2. FM patients have less variability of heart rate than healthy controls, as evidenced by diminished SDNN pNN50 RMSSD and SDSD parameters evaluated from the entire recording, as well as decreased SDNN (91.2 ms ± 21.5 versus 122.2 ms ± 32.0, *P *= 0.001), and SDANN (57.8 ms ± 19.2 versus 83.2 ms ± 35.9, *P *= 0.006) assessed during sleeping hours.

**Table 2:** Heart rate variability indexes in 22 female patients with fibromyalgia and 22 matched healthy controls.

	24-hour			Sleeping hours		
	
	Fibromyalgia	Controls	*P* value	Fibromyalgia	Controls	*P* value
Mean NN (ms)	758.4 ± 70.9	796.5 ± 92.0	0.132	881.5 ± 94.8	915.6 ± 122.9	0.310
SDNN (ms)	133.7 ± 28.7	152.6 ± 32.8	0.047	91.2 ± 21.5	122.2 ± 32.0	0.001
SDANN (ms)	120.0 ± 28.1	132.5 ± 27.9	0.144	57.8 ± 19.2	83.2 ± 35.9	0.006
SDSD (ms)	36.0 ± 13.9	47.3 ± 16.9	0.020	45.9 ± 19.6	56.9 ± 21.3	0.084
RMSSD (ms)	36.0 ± 13.9	47.3 ± 16.9	0.020	45.9 ± 19.6	56.9 ± 21.3	0.084
pNN50 (%)	13.6 ± 10.3	20.5 ± 11.6	0.045	23.9 ± 17.5	31.4 ± 17.5	0.160

**Figure 2:**
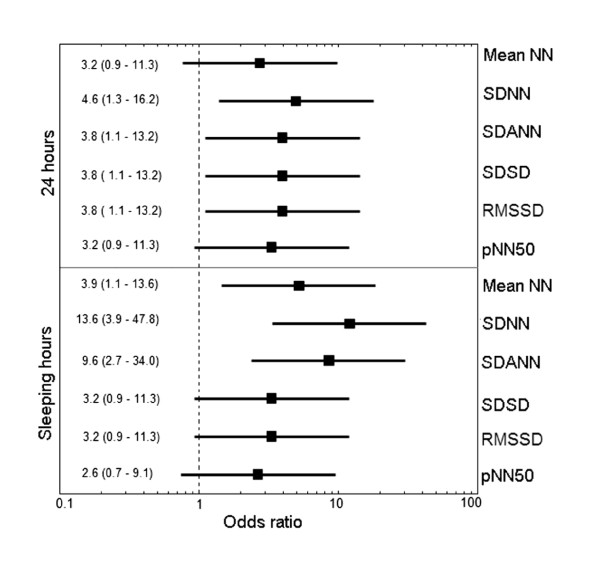
**Odds ratio of heart rate variability parameters evaluated from Holter recordings of 22 female patients with fibromyalgia and 22 matched healthy controls**. The cut-off points selected for each parameter are shown in Table 3. Data are presented as odds ratio (95% confidence interval)

Holter recordings were analyzed for 24 hours, and during sleeping time (from 00.00 to 06.00 hours). Results are shown as mean ± standard deviation. Student's *t *test calculated the differences between fibromyalgia group and control group. Mean NN, mean NN interval; ms, milliseconds; pNN50, percentage of adjacent pairs of R-R intervals that differ by more than 50 ms from each other in a given time period; RMSSD, Square root of the mean of the squares of differences between adjacent NN intervals; SDANN, mean standard deviation of the average NN intervals calculated over 5 minutes; SDNN, standard deviation of the NN intervals; SDSD, standard deviation of the successive NN differences;

Table 3 shows the quantitative Receiver Operating Characteristic (ROC)curve analysis of all HRV parameters. SDNN evaluated during sleeping hours yielded the best performance to set apart patients from controls. This observable fact is illustrated in Figure 2. SDNN odds ratio was 13.6 with 95% confidence intervals ranging from 3.9 to 47.8. Table 4 shows the predictive values of HRV indexes from the entire Holter recording and during sleeping hours (from 0000 to 0600). Nocturnal measurements have higher discriminating values. SDANN has the highest specificity (82) and highest positive predictive value (79) for FM diagnosis.

**Table 3:** Receiver-operator characteristic curve analysis of heart rate variability indexes

	AUC (95% CI)	*P*-value^a^	Best cut-off value	Distance to optimum value (0,1)
24-hour				

Mean NN (ms)	0.61 (0.45 - 0.79)	0.18	779	0.53
SDNN (ms)	0.67 (0.50 - 0.83)	0.05	141	0.45
SDANN (ms)	0.64 (0.48 - 0.81)	0.11	119	0.48
SDSD (ms)	0.69 (0.53 - 0.84)	0.03	40	0.48
RMSSD (ms)	0.69 (0.53 - 0.84)	0.03	40	0.48
pNN50 (%)	0.68 (0.52 - 0.83)	0.04	13	0.53

Sleeping hours				

Mean NN (ms)	0.59 (0.41 - 0.77)	0.30	922	0.49
SDNN (ms)	0.79 (0.66 - 0.92)	0.00	114	0.35
SDANN (ms)	0.75 (0.60 - 0.897)	0.00	59	0.37
SDSD (ms)	0.64 (0.48 - 0.81)	0.09	44	0.53
RMSSD (ms)	0.64 (0.48 - 0.81)	0.09	44	0.53
pNN50 (%)	0.63 (0.47 - 0.80)	0.13	31	0.55

^a^Null hypothesis is that area under the curve = 0.5.

Holter recordings were analyzed for 24 hours, and during sleeping time (from 00.00 to 06.00 hours). AUC, area under the curve; CI, confidence interval; Mean NN, mean NN interval; SDNN, standard deviation of the NN intervals; SDANN, mean standard deviation of the average NN intervals calculated over 5 minutes; ms, milliseconds; SDSD, standard deviation of the successive NN differences; RMSSD, square root of the mean of the squares of differences between adjacent NN intervals; pNN50, percentage of adjacent pairs of R-R intervals that differ by more than 50 ms from each other in a given time period.

**Table 4:** Predictive value of heart rate variability indexes in 22 patients with fibromyalgia and 22 controls.

	Sensitivity (%)	Specificity (%)	PPV (%)	NPV (%)
24-hour				

Mean NN ≤ 779 ms	73	55	62	67
SDNN ≤ 141 ms	68	68	68	68
SDANN ≤ 119 ms	64	68	67	65
SDSD ≤ 40 ms	64	68	67	65
RMSSD ≤ 40 ms	64	68	67	65
pNN50 ≤ 13%	55	73	67	62

Sleeping hours				

Mean NN ≤ 922 ms	73	59	64	68
SDNN ≤ 114 ms	86	68	73	83
SDANN ≤ 59 ms	68	82	79	72
SDSD ≤ 44 ms	55	73	67	62
RMSSD ≤ 44 ms	55	73	67	62
pNN50 ≤ 31%	68	55	60	63

Mean NN, mean NN interval; pNN50, percentage of adjacent pairs of R-R intervals that differ by more than 50 ms from each other in a given time period; ms, milliseconds; RMSSD, square root of the mean of the squares of differences between adjacent NN intervals; SDANN, mean standard deviation of the average NN intervals calculated over 5 minutes; SDNN, standard deviation of the NN intervals; SDSD, standard deviation of the successive NN differences.

**Figure 3:**
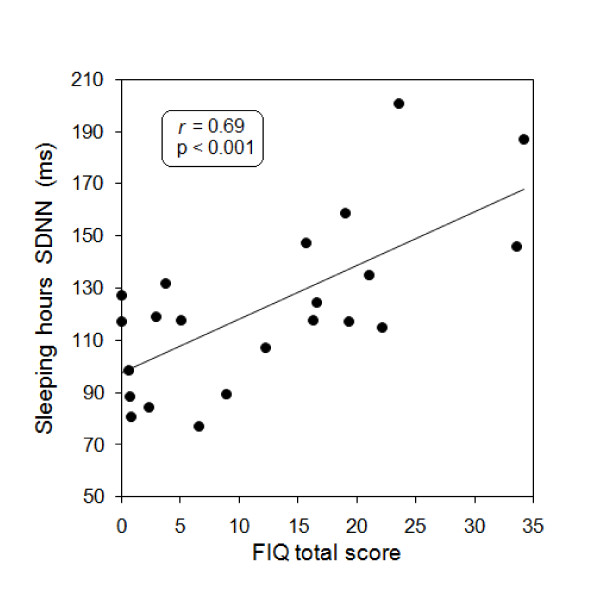
**Scatter gram of total FIQ scores and standard deviation of the NN intervals (SDNN) values evaluated during sleeping hours in 22 healthy controls**. The linear association was evaluated by Pearson's correlation coefficient (*r*). FIQ, fibromyalgia impact questionnaire.

There were multiple statistically significant associations between nocturnal HRV parameters and diverse FM symptoms. Table 5 displays only those significant correlations having an absolute *r *value ≥ 0.5. In the FM group, there is a correlation between HRV parameters indicative of sympathetic predominance and the severity of pain, constipation, and depression. In contrast, healthy controls display an opposite behavior. They have positive correlations between HRV markers indicative of parasympathetic predominance (either reduced sympathetic activity, increased vagal activity, or both) with the total FIQ score and the severity of fatigue, anxiety, and depression. Figure 3 shows a scatter gram of total FIQ scores and SDNN indexes in healthy controls during sleeping hours.

**Table 5:** Significant correlations with *r *absolute value ≥ 0.05 between symptom scales and HRV parameters evaluated during sleeping hours (0000 to 0600 hours).

	Fibromyalgia (n = 22)			Control (n = 22)		
	
		r	*P *value		r	*P *value
FIQ total score				SDNN	0.69	< 0.001
				SDANN	0.60	0.003
FIQ VAS pain	SDSD	-0.65	0.001			
	RMSSD	-0.65	0.001			
	pNN50	-0.62	0.002			
FIQ VAS fatigue				SDNN	0.65	0.001
				SDANN	0.57	0.005
FIQ VAS anxiety				SDNN	0.62	0.002
				SDANN	0.53	0.011
FIQ VAS depression				SDNN	0.52	0.012
				SDANN	0.51	0.015
COMPASS constipation	SDNN	-0.53	0.010			
HADS depression	Mean NN	-0.53	0.010			
SF-36 mental component	SDSD	0.64	0.001			
	RMSSD pNN50	0.64 0.64	0.001 0.001			

COMPASS, composite autonomic symptom scale; HADS, hospital anxiety and depression scale; FIQ, fibromyalgia impact questionnaire; Mean NN, mean NN interval; pNN50, percentage of adjacent pairs of R-R intervals that differ by more than 50 ms from each other in a given time period; RMSSD, square root of the mean of the squares of differences between adjacent NN intervals; SDANN, mean standard deviation of the average NN intervals calculated over 5 minutes; SDNN, standard deviation of the NN intervals; SDSD, standard deviation of the successive NN differences; SF-36, health survey short format 36 items; VAS, visual analogue scale.

## Discussion

The intense search for a valid FM biomarker has so far yielded unsatisfactory results. Dynamic neuroimaging techniques are able to distinguish FM patients from healthy controls [24] but these are expensive procedures and their distinctive features are not found in resting states but rather as a result of pain induction. HRV analysis is an economical non-invasive technique. Previous HRV studies in FM disclosed changes consistent with sympathetic predominance (summarized in [6]). Most studies were done during activity hours. We were able to find only one prior time-domain HRV investigation in FM focusing on nighttime measurements. Chervin *et al. *studied 13 patients with FM and 11 controls in a sleep laboratory. Similar to our results they found decreased nighttime SDANN in patients [25].

In the present investigation, nocturnal HRV parameters indicative of ongoing sympathetic predominance were able to clearly set apart FM patients from normal controls. It seems important to reiterate that sympathetic predominance means either higher sympathetic activity or decreased parasympathetic activity, or both. In the patient group HRV parameters suggestive of decreased parasympathetic activity correlated with different FM symptoms, including its main complaint: pain intensity.

HRV recording length strongly affects SDNN and SDANN. This is one reason why we did a fixed 00.00 hour to 06.00 hour sub-analysis. From the Holter tracings, we determined that participants were in bed during this part of the study. We did not perform a polysomnographic study, so we are not able to indicate if participants were asleep during this nighttime period.

There is an intimate relationship between autonomic performance and sleep. The HRV alterations found in FM patients probably reflect the lack of sustained deep restful sleep. However, the cause - effect connection of sleep, pain and autonomic dysfunction in FM needs further characterization.

Our study has several peculiarities: it was done in women while sleeping in the familiar environment of their homes rather than in a sleep laboratory. This home-based night-time appraisal allowed us to study individuals in a stable habitual basal situation in which most people are (or attempt to be) resting. None of the participants was on any medication that could alter autonomic activity. For this pilot study, we focused on women in the fertile period of their lives in order to avoid menopausal dysautonomic confounding variables.

Patients were sourced from several private rheumatology clinics in Mexico City. As a group they were lean (mean body mass index = 24.6 kg/m2) and greatly impacted by the FM (FIQ's mean value = 64 units). These characteristics probably do not apply to the FM population at large. It should be noted however, that controls had a similar age and a similar body mass index. Our database did not register variables that may affect HRV such as level of physical activity or use of oral contraceptives. It remains to be established if the HRV differences found in this study persist in obese post-menopausal women, in men or in patients with other painful rheumatic syndromes such as rheumatoid arthritis. In this regard, it is worth noting that a previous study revealed that FM patients have significantly more autonomic symptoms (including sleeping problems) than patients with rheumatoid arthritis [26]. Even so, a pending issue is to directly compare nighttime HRV parameters in FM versus other painful rheumatic syndromes.

Another novel finding of our study is the correlation between several nighttime HRV parameters indicative of sympathetic predominance and different FM symptoms, including pain severity. This correlation, observed in patients, does not define the cause-effect direction. Nevertheless, previous findings suggest that FM could be a sympathetically-maintained pain syndrome: patients who suffer from FM have norepinephrine-evoked pain [27], as well as COMT gene polymorphisms associated with a defective catecholamine clearing enzyme [9,10]. This body of evidence suggests that sympathetic hyperactivity could be the cause of FM symptoms.

An unexpected result of our study was the reverse correlation between HRV parameters and FM symptoms seen in healthy controls when compared to patients. We anticipated that there would be a continuum between HRV values and FM symptoms across these two groups. However, contrary to what is seen in patients, in healthy individuals, HRV markers suggestive of parasympathetic predominance have a positive correlation with some FM symptoms and with total FIQ scores. The reason for this surprising dichotomy is not clear. It could be speculated that healthy individuals have low basal sympathetic tone. For them, the feeling of fatigue or depression may be due to lack of sympathetic drive. In contrast, individuals with FM have higher basal sympathetic tone. The system is already overworked and exhausted. Further sympathetic activity is unable to overcome fatigue. In favor of this notion are different studies showing that in contrast to what is seen in healthy individuals, FM patients have blunted sympathetic responses to different types of stressors [6]. Another possible explanation for this dichotomy is that healthy individuals have harmonious circadian rhythms; thus, fatigue during the day is followed by a restful night.

Our results contradict the suggestion that FM is just one end of a continuous spectrum of 'normal' symptoms, including pain and fatigue [2,3]. Severe symptoms seen in patients have opposite autonomic correlations when compared to mild symptoms seen in healthy people. Based on previous studies from our institution [6], we propose that FM is a sympathetically-maintained neuropathic pain syndrome. Hypothetically, ongoing sympathetic hyperactivity, trauma, or infection could induce abnormal connections between the sympathetic nervous system and the nociceptive systems. These short-circuits may take place in the paravertebral nodules called dorsal root ganglia. Altered sodium channels may facilitate this pain sensitization. Sympathetic dysfunction can also explain non-pain related FM symptoms [28].

HRV analysis is a computer-based technology, therefore, it has abundant development potentials. Theoretically, the combined computerized nocturnal measurements of autonomic dependent vital sign variables, such as blood pressure, heart rate, and respiration, may yield even more discriminative values for FM. Because HRV analysis is a non-invasive technique, it may become a useful objective tool to estimate FM severity and/or responses to therapies.

## Conclusions

Nocturnal HRV parameters indicative of sympathetic predominance are significantly different in FM patients when compared to healthy individuals. In FM patients, there is a correlation between these HRV indices and several FM symptoms, including pain severity. Healthy controls display an opposite behavior. These results reinforce existing evidence suggesting that sympathetic hyperactivity could be the cause of FM symptoms. HRV parameters are potential FM biomarkers.

## Abbreviations

COMPASS: composite autonomic symptom scale; COMT: catechol-O-methyl- transferase; FIQ: fibromyalgia impact questionnaire; FM: fibromyalgia; HADS: hospital anxiety and depression scale; HRV: heart rate variability analysis; MAF: multidimensional assessment of fatigue; Mean NN: mean NN interval; MOS: medical outcome sleep scale; pNN50: percentage of adjacent pairs of R-R intervals that differ by more than 50 ms from each other in a given time period; RMSSD: square root of the mean of the squares of differences between adjacent NN intervals; SDANN: mean standard deviation of the average NN intervals calculated over 5 minutes; SDNN: standard deviation of the NN intervals; SDSD: standard deviation of the successive NN differences; SF-36: short format 36 items; VAS: visual analogue scale.

## Competing interests

The authors declare that they have no competing interests.

## Authors' contributions

CL and OI performed the HRV analyses and did the statistical analysis. AM, NR, and AV were involved in the clinical part of the investigation. MML conceived the study, participated in its design and helped to draft the manuscript. All authors made substantial contributions to the conception and design of the study. All authors read and approved the final version of the manuscript.

## References

[B1] Branco JC Bannwarth B Failde I Abello Carbonell J Blotman F Spaeth M Saraiva F Nacci F Thomas E Caubère JP Le Lay K Taieb C Matucci-Cerinic M Prevalence of fibromyalgia: a survey in five European countries Semin Arthritis Rheum 2010 39 448 453 10.1016/j.semarthrit.2008.12.003 19250656

[B2] Croft P Burt J Schollum J Thomas E Macfarlane G Silman A More pain, more tender points: is fibromyalgia just one end of a continuous spectrum? Ann Rheum Dis 1996 55 482 485 10.1136/ard.55.7.482 8774169 PMC1010214

[B3] Wolfe F Rasker JJ The Symptom Intensity Scale, fibromyalgia, and the meaning of fibromyalgia-like symptoms J Rheumatol 2006 33 2291 2299 16960921

[B4] Biomarkers Definitions Working Group Biomarkers and surrogate endpoints: preferred definitions and conceptual framework Clin Pharmacol Ther 2001 69 89 95 11240971 10.1067/mcp.2001.113989

[B5] Staud R Heart rate variability as a biomarker of fibromyalgia syndrome Fut Rheumatol 2008 3 475 483 10.2217/17460816.3.5.475 19890437 PMC2772072

[B6] Martinez-Lavin M Biology and therapy of fibromyalgia. Stress, the stress response system, and fibromyalgia Arthritis Res Ther 2007 9 R216 10.1186/ar2146 PMC2206360 17626613

[B7] Parati G Mancia G Di Rienzo M Castiglioni P Point: cardiovascular variability is/is not an index of autonomic control of circulation J Appl Physiol 2006 101 676 678 10.1152/japplphysiol.00446.2006 16645191

[B8] Huikuri HV Raatikainen MJ Moerch-Joergensen R Hartikainen J Virtanen V Boland J Anttonen O Hoest N Boersma LV Platou ES Messier MD Bloch-Thomsen PE Prediction of fatal or near-fatal cardiac arrhythmia events in patients with depressed left ventricular function after an acute myocardial infarction Eur Heart J 2009 30 689 698 19155249 10.1093/eurheartj/ehn537 PMC2655314

[B9] Vargas-Alarcón G Fragoso JM Cruz-Robles D Vargas A Vargas A Lao-Villadóniga JI García-Fructuoso F Ramos-Kuri M Hernández F Springall R Bojalil R Vallejo M Martínez-Lavín M Catechol-O-methyltransferase gene haplotypes in Mexican and Spanish patients with fibromyalgia Arthritis Res Ther 2007 9 110 10.1186/ar2324 17961261 PMC2212567

[B10] Barbosa FR Matsuda JB Mazucato M de Castro França S Zingaretti SM da Silva LM Martinez-Rossi NM Júnior MF Marins M Fachin AL Influence of catechol-O-methyltransferase (COMT) gene polymorphisms in pain sensibility of Brazilian fibromyalgia patients Rheumatol Int in press 10.1007/s00296-010-1659-z 21120493

[B11] Vargas-Alarcón G Fragoso JM Cruz-Robles D Vargas A Martinez A Lao-Villadóniga JI García-Fructuoso F Vallejo M Martínez-Lavín M Association of adrenergic receptor gene polymorphisms with different fibromyalgia syndrome domains Arthritis Rheum 2009 60 2169 2173 10.1002/art.24655 19565482

[B12] Moldofsky H Scarisbrick P England R Smythe H Musculosketal symptoms and non-REM sleep disturbance in patients with "fibrositis syndrome" and healthy subjects Psychosom Med 1975 37 341 351 169541 10.1097/00006842-197507000-00008

[B13] Wolfe F Smythe HA Yunus MB Bennett RB Bombardier C Glodenberg DL Tugwell P Campbell SM Abeles M Clark P Fam AG Faber SJ Fietchner JJ Frankling CM Gatter RA Hamaty D Lessard J Lichtbroun AS Maso AT McCain GA Reynolds J Romano TJ Russell IJ Sheon RP The American College of Rheumatology criteria for the classification of fibromyalgia: Report of the Multicenter Criteria Committee Arthritis Rheum 1990 33 160 172 10.1002/art.1780330203 2306288

[B14] Burckhardt CS Clark SR Bennett RM The fibromyalgia impact questionnaire: development and validation J Rheumatol 1991 18 728 733 1865419

[B15] Hays RD Stewart AL Stewart A, Ware J Sleep measures Measuring Functioning and Well-Being: the Medical Outcomes Study Approach 1992 Durham, NC: Duke University Press 235 259

[B16] Suarez GA Opfer-Gehrking TL Offord KP Atkinson EJ O'Brien PC Low PA The Autonomic Symptom Profile: a new instrument to assess autonomic symptoms Neurology 1999 52 523 528 10025781 10.1212/wnl.52.3.523

[B17] Zigmond AS Snaith RP The hospital anxiety and depression scale Acta Psychiatr Scand 1983 67 361 670 10.1111/j.1600-0447.1983.tb09716.x 6880820

[B18] Piper B Lindsey A Dodd M Ferketich S Paul S Weller S Funk S, Tornquist E, Champagne M, Wiese R The development of an instrument to measure the subjective dimension of fatigue Key aspects of comfort: Management of pain, fatigue, and nausea 1989 New York: Springer 199 207

[B19] Ware JE Jr Sherbourne CD The MOS 36-item short-form health survey (SF-36) Conceptual framework and item selection. Med Care 1992 30 473 483 1593914

[B20] Wessel N Voss A Malberg H Ziehmann Ch Voss HU Schirdewan A Meyerfeldt U Kurths J Nonlinear analysis of complex phenomena in cardiological data Herzschr Elektrophys 1990 11 159 173

[B21] Lerma C Infante O Perez-Grovas H Jose MV Poincaré plot indexes of heart rate variability capture dynamic adaptations after haemodialysis in chronic renal failure patients Clin Physiol Funct Imaging 2003 23 72 80 10.1046/j.1475-097X.2003.00466.x 12641600

[B22] Sollers JJ Buchanan TW Mowrer SM Hill LK Thayer JF Comparison of the ratio of the standard deviation of the R-R interval and the root mean squared successive differences (SD/rMSSD) to the low frequency-to-high frequency (LF/HF) ratio in a patient population and normal healthy controls Biomed Sci Instrum 2007 43 158 163 17487074

[B23] Martínez-Lavin M Hermosillo AG Rosas M Soto ME Circadian studies of autonomic nervous balance in patients with fibromyalgia. A heart rate variability analysis Arthritis Rheum 1998 42 1966 1971 10.1002/1529-0131(199811)41:11<1966::AID-ART11>3.0.CO;2-O 9811051

[B24] Dadabhoy D Crofford LJ Spaeth M Russell IJ Clauw DJ Biology and therapy of fibromyalgia. Evidence-based biomarkers for fibromyalgia syndrome Arthritis Res Ther 2008 10 211 10.1186/ar2443 18768089 PMC2575617

[B25] Chervin RD Teodorescu M Kushwaha R Deline AM Brucksch CB Ribbens-Grimm C Ruzicka DL Stein PK Clauw DJ Crofford LJ Objective measures of disordered sleep in fibromyalgia J Rheumatol 2009 36 2016 10.3899/jrheum.090051 PMC2909463 19684146

[B26] Solano C Martinez A Becerril L Vargas A Figueroa J Navarro C Ramos-Remus C Martinez-Lavin M Autonomic dysfunction in fibromyalgia assessed by the Composite Autonomic Symptoms Scale (COMPASS) J Clin Rheumatol 2009 15 172 176 10.1097/RHU.0b013e3181a1083d 19342959

[B27] Martinez-Lavin M Vidal M Barbosa RE Pineda C Casanova JM Nava A Norepinephrine-evoked pain in fibromyalgia. A randomized pilot study [ISRCTN70707830] BMC Musculoskelet Disord 2002 3 2 10.1186/1471-2474-3-2 11860612 PMC65524

[B28] Martinez-Lavin M Solano C Dorsal root ganglia sodium channels and fibromyalgia sympathetic pain Med Hypotheses 2009 72 64 66 10.1016/j.mehy.2008.07.055 18845401

